# Proposed criteria for the evaluation of the scientific quality of mandatory rat and mouse feeding trials with whole food/feed derived from genetically modified plants

**DOI:** 10.1007/s00204-016-1762-3

**Published:** 2016-06-21

**Authors:** Kerstin Schmidt, Janine Döhring, Christian Kohl, Maria Pla, Esther J. Kok, Debora C. M. Glandorf, René Custers, Hilko van der Voet, Jutta Sharbati, Ralf Einspanier, Dagmar Zeljenková, Jana Tulinská, Armin Spök, Clare Alison, Dieter Schrenk, Annette Pöting, Ralf Wilhelm, Joachim Schiemann, Pablo Steinberg

**Affiliations:** 1 BioMath GmbH, Schnickmannstr. 4, 18055 Rostock, Germany; 2 Institute for Food Toxicology and Analytical Chemistry, University of Veterinary Medicine Hannover, Bischofsholer Damm 15, 30173 Hannover, Germany; 3 Institute for Biosafety in Plant Biotechnology, Julius Kühn Institute (JKI), Federal Research Centre for Cultivated Plants, Erwin-Baur-Str. 27, 06484 Quedlinburg, Germany; 4 Edifici EPS1, Universitat de Girona (UDG), Campus Montilivi, 17071 Girona, Spain; 5 RIKILT Wageningen UR, Wageningen University and Research Centre, Akkermaalsbos 2, 6708WB Wageningen, The Netherlands; 6 GMO Office, National Institute for Public Health and the Environment/Centre for Safety of Substances and Products, Antonie van Leeuwenhoeklaan 9, 3721 MA Bilthoven, The Netherlands; 7 VIB (Flanders Institute for Biotechnology), Rijvisschestraat 120, 9052 Zwijnaarde, Belgium; 8 Wageningen University and Research Centre, Biometris, Droevendaalsesteeg 1, 6708PB Wageningen, The Netherlands; 9 Institute of Veterinary Biochemistry, Freie Universität Berlin, Oertzenweg 19b, 14163 Berlin, Germany; 10 Slovak Medical University, Limbová 12, 83303 Bratislava, Slovakia; 11 Alpen-Adria Universität Klagenfurt | Wien Graz, Schlögelgasse 2, 8010 Graz, Austria; 12 Roger Alison Ltd., Lampeter, SA48 8RN UK; 13 Food Chemistry and Toxicology, University of Kaiserslautern, 67663 Kaiserslautern, Germany; 14 Federal Institute for Risk Assessment, Max-Dohrn-Straße 8-10, 10589 Berlin, Germany

In recent years, animal feeding trials conducted with whole food/feed have been a focal issue in the controversy on the safety assessment of genetically modified (GM) plants and derived food/feed. Within the scientific community and among stakeholders, quite different views have been expressed on how these studies should be conducted, analysed and interpreted, what they might add in terms of information relevant to safety and whether 90-day rodent feeding trials should be mandatory. Despite the fact that the Commission Implementing Regulation (EU) No. 503/2013 (specifying the requirements for the risk assessment of GM food/feed) requests mandatory 90-day feeding trials for GM plants with single transformation events, the controversy continues. This is due to the fact that in 2016 the European Commission will have to review this particular provision in the legislation (ibid, Article 12), and because of questions raised by long-term feeding studies with GM maize.

In response to this controversy, the European Commission-funded project GRACE (GMO Risk Assessment and Communication of Evidence, www.grace-fp7.eu) conducted four 90-day feeding trials as well as a 1-year feeding trial with the GM maize MON810 and investigated the scientific value of animal studies for GMO risk assessment in comparison with alternative studies, i.e. those not being performed in animals.

In a similar approach, the current European Commission-funded project G-TwYST (GM Plants Two Year Safety Testing, www.g-twyst.eu) aims to inform GMO risk assessors (applicants, competent authorities) and policy makers on the possible added value of 90-day and extended animal feeding studies with whole food/feed. Moreover, it aims: (1) to clarify uncertainties raised through the outcomes and reports from recent (long-term) rodent feeding studies with the GM maize NK603; (2) to elaborate, if proven feasible, a scientifically sound approach for performing feeding studies with whole food/feed based on the OECD Test Guidelines 408 and 453 and subsequent EFSA recommendations. In order to pursue these aims, rat feeding trials with GM maize NK603 are being performed to test its potential subchronic toxicity, chronic toxicity and carcinogenicity.

In the context of the ongoing debate on GMO risk assessment in Europe, it is crucial to investigate: (1) criteria for evaluating the scientific quality of subchronic, chronic toxicity and carcinogenicity studies with whole food/feed in rats and mice; and (2) broader societal issues including ethical aspects associated with animal feeding trials in GM food/feed risk assessment. The former will help risk assessors in evaluating this type of studies when provided in the course of a pre-market risk assessment and will create a basis for further general debate. The latter will help to identify and better understand the specific challenges in the broader debate and how scientific aspects and normative dimensions are interrelated. Both aspects are also targeted by the G-TwYST project.

This letter specifically addresses the question on how to evaluate whole GM food/feed feeding trials. It does so by proposing a list of key quality criteria for the evaluation of 90-day and extended feeding trials with whole food/feed derived from GM plants. The proposed quality criteria should be taken into account when evaluating a feeding trial in the frame of an application to regulatory bodies and are not intended to be applied in other cases, in which, e.g. a feeding trial is performed to answer a specific open question in basic research.

In an EFSA explanatory statement, two possible risk assessment scenarios were described: scenario 1: relevant changes and/or specific hazards were identified in preceding analyses, and a feeding trial was planned and performed to test a specific hypothesis; scenario 2: no relevant changes and/or specific hazards were identified in preceding evaluations (i.e. no specific endpoints are targeted), and therefore, there is no specific hypothesis to be tested. Even though from a scientific point of view such scenario 2 feeding trials are not warranted when the GM plants show no substantial changes in composition and no indications of unintended effects, it is important to formulate quality criteria for these trials, as they have been made mandatory for GM food and feed by the Commission Implementing Regulation (EU) No. 503/2013. In both scenarios, the objective of a feeding trial is the detection of effects elicited by diets containing the whole GM food/feed, which could be of toxicological relevance. The present letter focuses on feeding trials falling under the EFSA scenario 2.

Based on the OECD Test Guidelines 408, 451, 452 and 453, the EFSA guidance on conducting repeated-dose 90-day oral toxicity study in rodents on whole food/feed, the EFSA considerations on the applicability of OECD TG 453 to whole food/feed testing and the EFSA explanatory statement on the above-mentioned EFSA guidance document, a number of issues to be considered when wanting to evaluate the quality of rat and mouse feeding trials performed for regulatory purposes with food/feed derived from GM plants were identified. As a result of extensive discussions on the different issues, a set of nine key quality criteria were identified and particular issues referring to the individual quality criteria were put together as described below. It is important to note that the described set of proposed key quality criteria applies to 90-day feeding trials as well as to long-term feeding trials such as those performed to test the potential chronic toxicity and/or carcinogenicity of whole food/feed with a duration of 12–24 months.

## Proposed criteria and issues related to each of them

1. The design of the feeding trial is based on internationally recognized test guidelines, but adapted for specific needs of whole food/feed studies and non-targeted testing.

There are a number of internationally recognized test guidelines, depending on the purpose and duration of the feeding trial, which can be recommended as starting points for the evaluation of whole food/feed. These are, among others, the OECD Test Guidelines 408 (90-day subchronic toxicity study), 451 (2-year carcinogenicity study), 452 (1-year chronic toxicity study) or 453 (combined chronic toxicity/carcinogenicity study), complemented by the EFSA guidance on conducting repeated-dose 90-day oral toxicity studies in rodents on whole food/feed, the EFSA considerations on the applicability of OECD TG 453 to whole food/feed testing and/or the EFSA explanatory statement on the above-mentioned EFSA guidance document. In the OECD documents, standardized testing protocols are described. However, since the OECD Test Guidelines were intended to evaluate chemicals, adaptations of these protocols are made when wanting to test whole food/feed.

The risk assessment scenarios 1 and 2 were originally described by EFSA for 90-day studies. In the case of scenario 2, in which no hypothesis to be tested has been identified, the study design based on the relevant OECD Test Guideline 408 and the corresponding EFSA recommendations is followed and the full range of observations/parameters described in the above-mentioned OECD Test Guideline is recorded. In the case of scenario 1, a study design of the feeding trial considering appropriate endpoints on a case-by-case basis will be required to test a specific hypothesis. In case of planning an extended feeding trial to test the potential chronic toxicity and/or carcinogenicity of whole food/feed derived from a GM plant, the protocols described in the corresponding OECD Test Guidelines are adapted, e.g. as described by EFSA.

Feeding trials with GM plants for regulatory purposes should preferably be performed following GLP principles. If certain parts of the study are conducted in compliance with GLP and others are not, this is clearly specified in the study report.

2. An analysis of the plant materials and diets including, among others, macro- and micronutrients, biological and chemical contaminants as well as the identification and quantification of the event, is performed.

A quantitative compositional analysis of the plant materials and diets is necessary to identify factors (e.g. the presence of a mycotoxin) other than the transgenic modification that could influence the outcome of the feeding trial. In case that such a factor is identified, the probability that it could lead to unintended effects in the laboratory animals is considered when evaluating the outcome of the feeding trial. The analysis of the plant material and diets includes nutrients [proximates (ash, total carbohydrates, fat, protein), starch, fibres, fatty acids, amino acids, sugars, minerals, vitamins], and plant-specific secondary metabolites, in particular antinutrients (e.g. phytic acid, trypsin inhibitor, lectins) and toxicants as specified in the OECD Consensus documents for the work on the Safety of Novel Foods and Feeds: Plants (http://www.oecd.org/science/biotrack/consensusdocumentsfortheworkonthesafetyofnovelfoodsandfeedsplants.htm). In addition, the plant material and diets are tested for the presence of genetically modified organisms, chemical contaminants (heavy metals, nitrosamines, polychlorinated dioxins, polychlorinated biphenyls, polycyclic aromatic hydrocarbons, pesticides, mycotoxins) and the microbial contamination. Representative plant materials and diet samples are taken for the analyses. Based on the analysis of the plant material, nutritionally balanced diets adjusted to the dietary requirements of the rat or mouse strain used are formulated.

The identity of the genetic event in the plant materials and diets as well as the presence of other events as background contamination in the whole food/feed is documented. Moreover, depending on the availability of an established method, the levels of the protein encoded by the transgene are quantified.

3. The highest level of the plant material that can be incorporated in the animal diets without leading to a nutritional imbalance is tested.

The plant material is fed orally and ad libitum. In principle, it is not necessary to use two dose levels in addition to the control (zero dose level), but in practice two dose levels may help in the interpretation/evaluation of results in order to distinguish between treatment- and non-treatment-related effects.

EFSA suggested the following incorporation rates as reference values for high doses in 90-day feeding trials in rodents: 60 % for rice (dehulled), 50 % for maize, 30 % for soybean meal, 25 % for rapeseed meal and 20 % for full fat soybean as well as potatoes (heated, dried). In this regard, a preliminary “maximal inclusion rate finding study” to determine the maximal level of material from a specific plant species that could be incorporated in the animal diet without leading to a nutritional imbalance is conducted before performing a 90-day feeding trial, particularly in case of an incorporation rate or a plant species never having been tested before in a feeding trial.

When wanting to test the potential long-term effects (chronic toxicity and/or carcinogenicity) of whole food/feed derived from GM plants, one has to take into account whether the whole food/feed incorporation rate, independently of the event, might lead to any organ alteration on the long-term (i.e. within 12–24 months) due to the increased concentration of a normal plant constituent in the food/feed. In such a case, the high incorporation rate is reduced when compared to that used in the 90-day feeding trials.

4. A non-GM line with a comparable genetic background is used as a control.

A non-GM line with a comparable genetic background (i.e. near-isogenic in the case of sexually propagated crops, the non-GM isogenic variety in the case of vegetatively propagated crops) is used as a control. The control line has a well-established history of safe use. Moreover, the control group receives the control material at the same incorporation rate used for the high-dose group. If intermediate dose groups are included, the test material is supplemented with the control material in order to achieve the same total incorporation rate.

5. Specific aspects regarding the choice and housing of the laboratory animals used in the feeding trials are considered.

A justification for the chosen animal species (rat, mouse) is given. Both sexes of the chosen laboratory animals are used. In the case of rats (but not in the case of mice), animals of the same sex are housed in pairs. The laboratory animals are housed in an animal housing facility under controlled conditions (e.g. room temperature: 22 ± 3 °C; humidity: 40–70 %; 12/12 h day and night cycle). Animals are acclimatized to the animal housing facility conditions for at least 5 days prior to the start of the study and are not subjected to any previous experimental procedures. Animals are uniform in age, with minimal body weight variation at the beginning of the feeding trial (i.e. ±20 % of the mean weight of each sex), and the age of the animals at the beginning of the feeding study is not greater than 9 weeks.

6. Appropriate randomization techniques are applied.

Animals are assigned to the different experimental groups by randomization. In this context, a randomized blocking, which allows to control for background variations (body weight, position of the animal in the room), is performed. Furthermore, where possible, the randomized blocking should be extended to the analysis phase (e.g. weighing of the animals, dissection of the animals, biochemical analysis of blood and urine are to be done block by block). The cage is used as the experimental unit and considered part of the experimental design and sampling strategy.

7. A reliable and appropriate sample collection and processing strategy is implemented.

Body weight and feed consumption are monitored regularly. Although cage mean values are used for the statistical analysis (since the cage is the experimental unit), the individual animal body weight values are reported if a cage houses two animals.

The fasting period prior to blood sampling is the same for all animals. This is an important point to decrease variability in the haematology and clinical biochemical analyses. Rats are fasted overnight (i.e. about 16 h), in the case of mice the period should only comprise 5–6 h. Blood is taken from the same site in all animals, and possible deviations are justified. Blood is stored under appropriate conditions, either without or with an anticoagulant as specified.

The clinical chemistry analyses of the blood samples are completed as quickly as possible, ideally within 1 day, to minimize potential variability between the samples. An analysis within 1 day might not be possible when handling a high number of samples, as occurs in the case of feeding trials being performed according to internationally accepted guidelines. In such a case, the analyses are performed according to a randomized sampling design (see criterion no. 6), where all analyses within one “sampling block” are done closely in time. The date of the analyses is recorded, and the sampling structure is considered when performing the statistical analyses.

Animals are necropsied in the order fixed in the randomized block design of the experiment (see criterion no. 6), i.e. the same randomization scheme applied at the beginning when assigning animals to the different experimental groups is applied for the gross necropsy.

8. The staff performing the feeding trial and the analysis of the plant materials, diets and animal samples is “blind” with respect to the identity of the diets.

The advantage of a blind study is that it avoids the influence of any bias that experimenters and analysts may have with regard to possible outcomes. Without blinding, prior expectations may lead to an unconscious biasing of actions and observations. This is particularly relevant for those endpoints in which subjective elements might influence the observations. The blinding is applied to all staff involved, such as the personnel working with the animals, those that weigh the animals, those involved in the necropsy, and all analyses of material stemming from the experiments.

The dose groups are only unblinded for the histopathological evaluation of the tissues after necropsy and the weighing of organs. This means that in a first instance the tissues/organs are evaluated histopathologically in an unblinded manner, and in case of suspected treatment-related findings, a blinded re-evaluation is performed.

9. Appropriate statistical methods are applied to evaluate the power of the study and to analyse the obtained results.

A preliminary power analysis is performed to estimate the probability to detect effect sizes of potential relevance. Effect sizes of potential relevance are based on an expert opinion, but may also be inspired by the analysis of datasets from non-GM groups in previous studies with the same plant species and with sufficient similarity to the intended animal study. The power analysis requires for all parameters the specification of: (a) the significance level of the test (this will be assumed to be 5 %, unless regulatory requirements justify another value); (b) the sample size (which will be that of the intended design); (c) the effect size (this will be the effect size of potential relevance); and (d) the variability, e.g. the standard deviation (within each dose group) of the measurements (this will have to be derived from previous data or be specified based on an expert opinion). In a scenario 2, standard sample sizes (e.g. mentioned in OECD Test Guidelines) are used. The role of a power analysis is then to check the statistical power of tests for the different parameters to be measured. The power analysis may focus on a subset of endpoints of primary interest.

In the frame of the statistical analysis, group differences (effect sizes) of interest (e.g. GM group vs. control group) are tested for effects different from zero (difference tests). Finally, toxicologists assess whether the observed effects are relevant from a toxicological point of view by taking into account the complete set of measured parameters, whether or not the differences are significantly different.

All statistical methods are reported and comprehensively described. It is important to differentiate between the “classical” (analysis of variance + post hoc test) and novel approaches (e.g. standard effect size analyses) until the risk assessment community has accepted the latter approaches. The statistical analysis plan is explicit regarding what mistakes/shortcomings should be avoided. Furthermore, raw data are made available to third parties to allow for an independent analysis.

## Concluding remarks

Taken together, nine criteria to evaluate the scientific quality of rat and mouse feeding trials with whole food/feed derived from genetically modified plants are proposed and a number of specific aspects to be taken into account in conjunction with these individual quality criteria are addressed. It is recommended that the quality assessment of a feeding trial in the frame of a regulatory decision process is made on a case-by-case basis considering all relevant quality criteria proposed in this letter. It is important to note that a feeding trial does not automatically provide useful information simply because it meets the nine proposed criteria.

Only in case a trigger is available from the initial molecular, compositional, phenotypic and/or agronomic analyses and therefore the rationale of the study prior to testing is formulated in form of hypotheses regarding specific endpoints, feeding trials with whole food/feed may provide an added scientific value for the risk assessment of GM crops.

It is expected that this letter will trigger a broader scientific debate on the quality of rodent feeding trials with whole food/feed, and for this purpose, contributions are welcome in the Discussion Forum established by the scientific coordinators of the European research projects GRACE, G-TwYST and GMO90+ in *Archives of Toxicology.*

